# An evaluation of the timing of surgical complications following radical prostatectomy: Data from the American College of Surgeons National Surgical Quality Improvement Program (ACS-NSQIP)

**DOI:** 10.1080/2090598X.2020.1749478

**Published:** 2020-04-17

**Authors:** Ali Merhe, Nassib Abou Heidar, Mohamad Hout, Gerges Bustros, Aurelie Mailhac, Hani Tamim, Wassim Wazzan, Muhammad Bulbul, Rami Nasr

**Affiliations:** a Department of Surgery, Division of Urology, American University of Beirut Medical Center, Beirut, Lebanon; b American University of Beirut Medical Center, Faculty of Medicine, Biostatistics and Clinical Research Unit, Beirut, Lebanon

**Keywords:** Timing of complications, prostatectomy, length of stay, mortality

## Abstract

**Objective:**

To perform a time-to-complication analysis for radical prostatectomy (RP) and computing risk factors for these complications, as RP is established as a first-line treatment for localised prostate cancer with excellent oncological outcomes but is not without its complications.

**Patients and methods:**

We used the National Surgical Quality Improvement Program (NSQIP) database to analyse data of patients who underwent RP, between 2008 and 2015, with the primary endpoint of time-to-complications. Categorical variables were analysed using descriptive statistics and continuous variables were recorded as medians and interquartile ranges (IQRs) such as timing of complications. Multivariable regression analyses were used to analyse time-to-complication and its effect on other outcomes. A *P* < 0.05 was defined as statistically significant.

**Results:**

The overall 30-day complication rate was 7.54% and was equally distributed before and after discharge. Bleeding/transfusion (3.37%), urinary tract infection (1.58%), deep venous thrombosis (DVT; 0.74%), and wound infection (1.08%) were the five most common complications after RP. The median (IQR) time-to-complication unique for each complication was: bleeding/transfusion occurred on the same operative day (1), renal complications occurred at 4 (2–6) days, sepsis at 12 (6.5–17.5) days, DVT at 11 (5.5–16.5) days, pneumonia at 4 (0.5–7.5) days, and cardiac arrest occurred at 5 (1.75–8.25) days. After discharge complications were associated with greater odds of re-admission (odds ratio [OR] 16.40, *P* < 0.001), but associated with a lesser length of stay (OR – 3.33, *P* < 0.001) when compared to pre-discharge complications.

**Conclusion:**

Several risk factors predict pre- and post-discharge complication rates. Knowledge regarding the timing of complications and their respective risk factors should improve patient–physician communication and prediction, and thus patient care.

**Abbreviations:**

ACS: American College of Surgeons; BMI: body mass index; DM: diabetes mellitus; DVT: deep venous thrombosis; Hct: haematocrit; IQR: interquartile range; LOS: length of stay; NSQIP: National Surgical Quality Improvement Program; OR: odds ratio; RP: radical prostatectomy

## Introduction

Prostate cancer is the most common cancer in males and one of the leading causes of cancer mortality worldwide [1,2]. Radical prostatectomy (RP) is established as a first-line treatment for localised prostate cancer, with excellent oncological outcomes [3]. Despite this, the RP procedure can lead to many complications. Hu et al. [4] evaluated Medicare-linked Surveillance, Epidemiology, and End Results (SEER) data and found the unadjusted rate of overall complications was ~23%, regardless of approach. Many studies have addressed the rate of complications, with many variations in the rates and their possible risk factors [5–7]; however, no study has addressed the timing of complication occurrence. Knowledge of the timing of a complication aids in anticipation of its occurrence and helps the implementation of measures to prevent it from happening and prevent its sequelae [8,9]. We believe that the identification of the patients at risk of pre- and post-discharge adverse events would pave the way for better patient counselling about RP and its outcomes.

## Patients and methods

We utilised data from the American College of Surgeons National Surgical Quality Improvement Program (ACS-NSQIP) to identify the median time-to-event data for principal postoperative complications within 30 days of RP, as well as the predictors of pre- and post-discharge complications, and lastly we studied the effect of timing of complications on secondary adverse events including length of stay (LOS), rate of re-intervention, rate of re-admission, and 30-day mortality.

The ACS-NSQIP database contains risk adjustable surgical patient data from different participant hospitals. Trained surgical clinical reviewers prospectively collected ACS-NSQIP data and validated it from patients’ medical records allowing quantification of 30-day surgical outcomes. Patients were identified in the ACS-NSQIP (2008–2015) using Current Procedural Terminology (CPT) codes for RP (55810, 55840, 55841, 55845, and 55866) with a principal postoperative diagnosis of prostate cancer (International Classification of Diseases [ICD] 9 code 185).

Overall, 36 753 patients were available for analysis. For each patient, age; body mass index (BMI); race; smoking status; comorbidities, including hypertension, diabetes mellitus (DM), cardiopulmonary disease; preoperative haematocrit (Hct); and preoperative creatinine; were recorded. The primary endpoint was time-to-complication (pre- vs post-discharge). Complications including: superficial, deep and organ/space surgical site infection, wound dehiscence, pneumonia, unplanned intubation, pulmonary embolism, need for ventilator support for >48 h, progressive renal insufficiency, acute renal failure, UTI, myocardial infection, cardiac arrest requiring cardiopulmonary resuscitation, bleeding/transfusion, deep venous thrombosis (DVT)/thrombophlebitis, sepsis, and septic shock, were all reported. The overall complication rate was defined as the occurrence of any complication. Secondary endpoints were the LOS, re-intervention, re-admission, and perioperative mortality. The median time-to-event was recorded for each complication individually from the date of RP and then in relation to discharge. Categorical variables were analysed by descriptive statistics and analysed in the form of frequencies, as well as proportions. Whilst, continuous variables were recorded as medians and interquartile ranges (IQRs). A chi-square test was used to compare proportions. Multivariable regression analyses were used to analyse time-to-complication and its effect on other outcomes. The statistical analysis was performed using the Statistical Package for the Social Sciences (SPSS®), version 24.0 (SPSS Inc., IBM Corp., Armonk, NY, USA), and a two-sided *P* < 0.05 was defined as statistically significant. Due to the nature of the database, the study was exempt from the Institutional Review Board.

## Results

Table 1 provides descriptive characteristics of 36 753 patients who underwent RP during the study period. The median (IQR) age of the cohort was 62 (55.2–70) years. Most patients were White (84%). In all, 38% of the patients were obese and 12.5% were active smokers. Hypertension appeared to be the most common comorbidity (52.96%), followed by DM (12.6%) and cardiopulmonary disease (2.01%). These comorbidities were more common in patients who developed complications (*P* < 0.001). Most patients had normal renal function (83.1%). The median LOS was 1.8 days and the overall 30-day complication rate was 7.54% (2722 of 36 753 patients), with 1497 (55%) patients having pre-discharge complications and 1275 (46.8%) post-discharge complications. Bleeding/transfusion (3.37%), UTI (1.58%), DVT (0.74%), and wound infection (1.08%) were the five most common complications after RP.

**Table 1.** Characteristics of the included patients undergoing RP stratified by complication status.

Characteristic	No complications (*N* = 33981)	Pre-discharge complications (*N* = 1497)	Post-discharge complications (*N* = 1275)	*P*
Age, years, mean (SD)	62.4 (7.2)	64.2 (8.2)	62.7 (7.5)	<0.001
*N* (%)				
BMI, kg/m^2^ <18.5 18.5–24.9 25–29.9 ≥30	102(0.3) 5829 (17.15) 15267 (44.93) 12783 (37.62)	13 (0.87) 304 (20.31) 660 (44.09) 520 (34.74)	5(0.39) 157 (12.31) 507 (39.76) 606 (47.53)	<0.001
Race White Black Other	28547 (84.01) 4355 (12.82) 1079 (3.18)	1294 (79.8) 265 (16.4) 62 (3.8)	1205 (83.6) 206 (14.3) 31 (2.1)	<0.001
Smoker	4242 (12.48)	207 (13.83)	180 (14.12)	0.077
Hypertension	17998 (52.96)	910 (60.79)	700 (54.9)	<0.001
DM	4281 (12.6)	258 (17.23)	201 (15.76)	<0.001
Cardiopulmonary*	684 (2.01)	49 (3.27)	40 (3.14)	<0.001
Preoperative creatinine, mg/dL <1.2 ≥1.2	28238 (83.1) 5743 (16.9)	1136 (75.89) 361 (24.11)	1021 (80.08) 254 (19.92)	<0.001
Preoperative Hct, % <30 30–45 >45	121 (0.36) 23766 (69.94) 10094 (29.7)	59 (3.94) 1149 (76.75) 289 (19.31)	8 (0.63) 893 (70.04) 374 (29.33)	<0.001

*cardiopulmonary comorbidities include patients with history of myocardial infarction, coronary artery disease, and chronic obstructive pulmonary disease.

**Figure 1.**
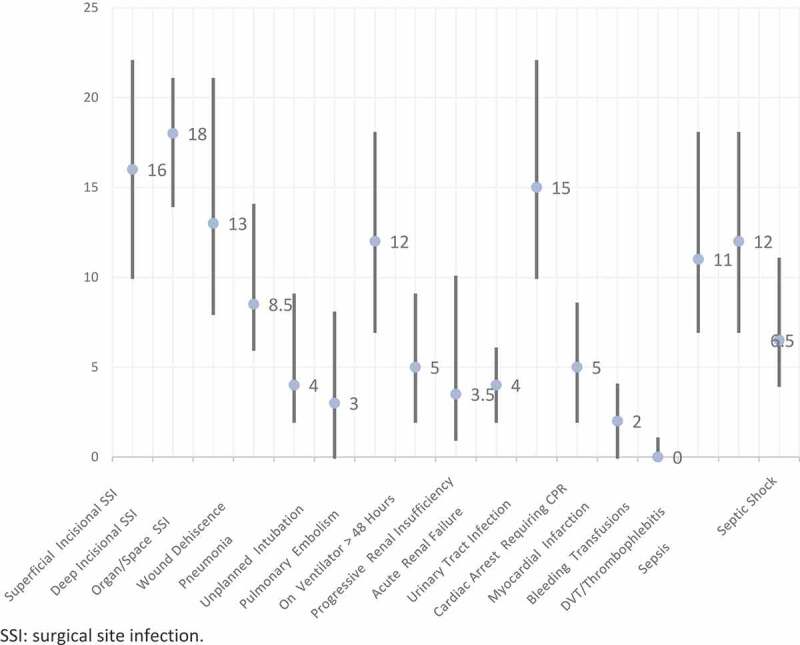
Time-to-complication at ≤30 days of RP (number indicating median days and bars indicating interquartile range) amongst the 36 753 patients. SSI: surgical site infection.

**Figure 2.**
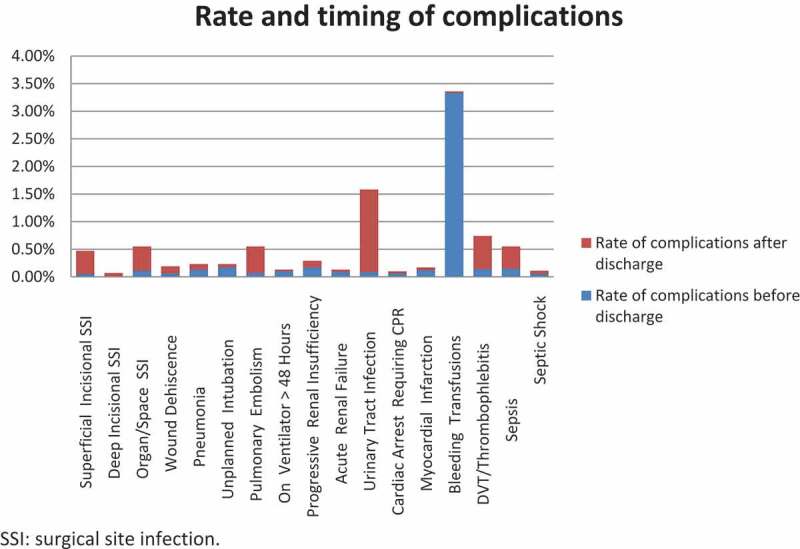
Proportion of complications amongst RP patients occurring before and after discharge. SSI: surgical site infection.

The median time-to-event for the studied complications is shown in Figure 1. The median (IQR) time-to-complications unique for each complication was: bleeding/transfusion occurred on the same operative day (1), renal complications occurred at 4 (2–6) days, sepsis at 12 (6.5–17.5) days, DVT at 11 (5.5–16.5) days, pneumonia at 4 (0.5–7.5) days, and cardiac arrest at 5 (1.75–8.25) days. The median time to relatively minor complications, including UTI and wound infection was 15 and 16 days, respectively. Overall, 46% of complications occurred after discharge. Figure 2 shows the proportion of complications that occurred before and after discharge. Most UTIs occurred after discharge (1.49%), while 0.09% occurred before discharge. Bleeding and pulmonary complications tended to occur more frequently before discharge.

In multivariable analysis (Table 2), age was a significant predictor of both pre- and post-discharge complications [odds ratio (OR) 1.026, *P* < 0.001; and OR 1.009, *P* = 0.028 respectively]. Being underweight, i.e., BMI <18.5 kg/m^2^, significantly increased the risk only of pre-discharge complications (OR 1.936, *P* = 0.034). Patients with a BMI >25 and >30 kg/m^2^ had a significantly lower risk of pre-discharge (OR 0.857, *P* = 0.033) but a higher risk of post-discharge events (OR 1.245, *P* = 0.019) when compared to normal-weight patients. Black race patients had a higher risk of pre-discharge complications when compared to the White race (OR 1.206, *P* = 0.014); however, this was not significant for post-discharge events. Smoking had no significant effects on either pre- or post-discharge events. Patients who had hypertension preoperatively were more likely to develop pre-discharge complications only (OR 1.202, *P* = 0.001). While those who had DM were more likely to have pre- and post-discharge complications (OR 1.199, *P* = 0.014; and OR 1.180, *P* = 0.044, respectively). Patients with cardiopulmonary diseases had significantly higher odds of post-discharge complications (OR 1.462, *P* = 0.024). When compared to patients with normal Hct, patients with a preoperative Hct <30% had a significantly higher risk of pre-discharge complications (OR 8.176, *P* < 0.001), in contrast to those with Hct >45% that had a lower odds of pre-discharge events (OR 0.639, *P* < 0.001). Preoperative chronic renal disease increased the risk of pre-discharge complications (OR 1.294, *P* < 0.001)

**Table 2.** Multivariable logistic regression analysis of predictors of timing of complications with respect to discharge.

Multivariate – predictor				
*N* = 36 753				
Variable	Pre-discharge complications	*P*	Post-discharge complications	*P*
Age	1.026 (1.018–1.034)	<0.001	1.009 (1.001–1.017)	0.028
BMI, kg/m^2^				
<18.5	1.936 (1.052–3.561)	0.034	1.599 (0.640–3.994)	0.315
18.5–24.9	1 (Ref)		1 (Ref)	
25–29.9	0.857 (0.743–0.988)	0.033	1.245 (1.036–1.495)	0.019
≥30	0.778 (0.668–0.906)	0.001	1.763 (1.467–2.119)	<0.001
Race				
White	1 (Ref)		1 (Ref)	
Black	1.206 (1.039–1.401)	0.014	1.103 (0.935–1.302)	0.244
Other	1.111 (0.844–1.463)	0.453	0.619 (0.407–0.941)	0.024
Smoker				
No	1 (Ref)		1 (Ref)	
Yes	1.133 (0.968–1.326)	0.119	1.177 (0.997–1.390)	0.054
Hypertension				
No	1 (Ref)		1 (Ref)	
Yes	1.202 (1.073–1.347)	0.001	0.923 (0.819–1.041)	0.193
DM				
No	1 (Ref)		1 (Ref)	
Yes	1.199 (1.036–1.387)	0.014	1.180 (1.004–1.386)	0.044
Cardiopulmonary				
No	1 (Ref)		1 (Ref)	
Yes	1.310 (0.965–1.779)	0.083	1.462 (1.050–2.035)	0.024
Preoperative creatinine, mg/dL				
<1.2	1 (Ref)		1 (Ref)	
≥1.2	1.294 (1.138–1.470)	<0.001	1.154 (0.999–1.333)	0.052
Preoperative Hct, %				
<30	8.176 (5.909–11.313)	<0.001	1.599 (0.777–3.290)	0.203
30–45	1 (Ref)		1 (ref)	
>45	0.639 (0.560–0.730)	<0.001	0.995 (0.879–1.127)	0.941

Table 3 lists the effect of complication timing on secondary endpoints. A pre-discharge complication increased the odds of re-intervention (OR 2.93, *P* < 0.001), re-admission (OR 19.16, *P* < 0.001), mortality (OR 24.58, *P* < 0.001), and prolonged the LOS (OR 3.97, *P* < 0.001). Similarly, a post-discharge complication also resulted in requirement for re-intervention (OR 47.59, *P* < 0.001), re-admission (OR 15.53, *P* < 0.001), mortality (OR 24.00, *P* < 0.001), and prolonged the LOS (OR 0.41, *P* < 0.001). When the multivariable analysis was done for the timing of complications, post-discharge complications were associated with greater re-admission odds (OR 16.40, *P* < 0.001), and post-discharge complications were associated with a lesser LOS (OR – 3.33, *P* < 0.001) when compared to pre-discharge complications.

**Table 3.** (a) Multivariable regression analysis studying effect on pre- and post-discharge complications on re-intervention, re-admission, LOS and mortality (no complication taken as reference) and controlling for age, gender, BMI, race, smoking, creatinine, comorbidities, and haematocrit. (b) Multivariable regression analysis evaluating the effect of only timing on the same secondary outcomes (pre-discharge complication reference) while controlling for the same variables.

		Re-admission related		Re-operation related	Mortality		LOS
**A**							
Patients without complications		Reference		Reference	Reference		Reference
Pre-discharge complications	OR (95% CI)	2.93 (2.27–3.77)		19.16 (14.84–24.75)	24.58 (12.31–40.05)	Β (95% CI)	3.97 (3.85–4.1)
	*P*	<0.001		<0.001	<0.001	*P*	<0.001
Post-discharge complications	OR (95% CI)	47.59 (41.10–55.12)		15.53 (11.60–20.79)	24.00 (11.81–48.79)	Β (95% CI)	0.41 (0.28–0.54)
	*P*	<0.001		<0.001	<0.001	*P*	<0.001
**B**							
Pre-discharge complications			Reference	Reference	Reference		Reference
Post-discharge complications	OR (95% CI)		16.40 (12.53–21.46)	0.82 (0.61–1.12)	0.94 (0.47–1.91)	Β (95% CI)	–3.33 (–3.73 to – 2.93)
	*P*		<0.001	0.21	0.87	*P*	<0.001

*For the LOS outcome a univariate general linear model was constructed with the covariates as described above, whereas for other variables, a logistic regression model was used.

## Discussion

RP is the preferred option for treating certain intermediate- and high-risk localised prostate cancer [10]. However, time-to-complication data are lacking among patients undergoing RP. These data are crucial, as they may give the treating physician, as well as the patient, a better understanding of the postoperative course, as well as timely prediction of possible complications in the perioperative period. In the present study, we addressed this issue using the largest multi-institutional national surgical database. The present study, to our best knowledge, is unique in the urological literature and reflects real-life outcomes from many institutions with variable surgical expertise.

Several important findings can be highlighted in our present study. Most importantly, the overall complication rate for RP was 7.5%, with complications occurring almost equally before and after discharge. Pulmonary and bleeding complications tended to occur more often before discharge, while UTIs and thromboembolic events tended to occur more frequently after discharge.

Moreover, we identified risk factors associated with the timing of complications and found that these risk factors were distinct. For instance, we found that age, hypertension, DM, BMI <18.5 kg/m^2^, and preoperative anaemia (Hct <30%) were independent predictors of pre-discharge complications. Whereas age, BMI ≥30 kg/m^2^, and pre-existing cardiopulmonary disease were predictors of post-discharge complications.

These data provide ample information for the physician to provide a more detailed discussion with the patient undergoing RP before surgery and even before discharge, thus potentially improving patient satisfaction [11]. Also, this information can be used to stratify risk profiles of the patient to better help allocate resources in terms of increased vigilance in the early postoperative course [12,13] or more patient-tailored follow-up appointments of patients after RP [14]. However, more studies are required to identify the magnitude of the complications and their respective risk factors, so that these can be better anticipated.

These data show that the timing of complications does not influence mortality or the need for re-intervention, but does increase the risk of re-admission. This contradicts the findings of Wakeam et al. [15] that link the timing of complications in all in-patient surgeries with postoperative mortality. This difference could be attributable to our focus on RP rather than all in-patient surgeries. Furthermore, our present data validate previous reports that indicate that pre-discharge complications are associated with a more extended hospitalisation, while post-discharge complications increase the odds for re-admission [16–18]. We could deduce from the present study that post-discharge complications are mainly infectious and not life-threatening, which is why they increase re-admissions but not mortality.

The present study highlights an important point as well; the classic rule of W’s does not always apply (Wind, Water, Walk, Wound, Wonder Drugs). For instance, thromboembolic complications tended to occur before UTIs. The challenge of this paradigm taught in medical schools is not new. However, it has been challenged by Sood et al. [14,18] in previous similar studies pertaining to nephrectomies and radical cystectomies.

The present study is not without its own limitations. For instance, prostate cancer stage and grade, socioeconomic variables, whether the RP was primary or salvage, as well as hospital and surgeon volume, are not available in the ACS-NSQIP. Our present study does not account for interaction between pre- and post-discharge complications in the same patient. Furthermore, the complications are not stratified in the ACS-NSQIP in terms of severity and procedure-specific complications, such as anastomotic urine leak or anastomotic strictures. Moreover, the complication grade is not reported as per any formal classification such as the Clavien–Dindo classification, which might affect the quality of the present study. Despite the mentioned limitations, the present study of the ACS-NSQIP data is the first to examine the time-to-complication following RP at a multi-institutional level.

## Conclusion

Complication rates following RP were ~7.5%, and these were evenly split before and after discharge. Several risk factors predicted pre- and post-discharge complication rates that can be used in the future to predict outcomes, such as pre-procedure medical state (hypertension, DM, and cardiopulmonary diseases). Accordingly, properly predicting timing of events can better help manage patient expectations, and perform patient-specific follow-up schedules.

## References

[1] Azubuike SO, Muirhead C, Hayes L, et al. Rising global burden of breast cancer: the case of sub-Saharan Africa (with emphasis on Nigeria) and implications for regional development: a review. World J Surg Oncol. 2018;16:63. 29566711 10.1186/s12957-018-1345-2 PMC5863808

[2] Arnold M, Karim-Kos HE, Coebergh JW, et al. Recent trends in incidence of five common cancers in 26 European countries since 1988: analysis of the European cancer observatory. Eur J Cancer. 2015;51:1164–1187. 24120180 10.1016/j.ejca.2013.09.002

[3] Heidenreich A, Bastian PJ, Bellmunt J, et al. EAU guidelines on prostate cancer. part 1: screening, diagnosis, and local treatment with curative intent-update 2013. Eur Urol. 2014;65:124–137. 24207135 10.1016/j.eururo.2013.09.046

[4] Hu JC, Gu X, Lipsitz SR, et al. Comparative effectiveness of minimally invasive vs open radical prostatectomy. JAMA. 2009;302:1557–1564. 19826025 10.1001/jama.2009.1451

[5] Pompe RS, Beyer B, Haese A, et al. Postoperative complications of contemporary open and robot-assisted laparoscopic radical prostatectomy using standardised reporting systems. BJU Int. 2018;122:801–807. 29727912 10.1111/bju.14369

[6] Hiess M, Ponholzer A, Lamche M, et al. The Clavien-Dindo classification of complications used for radical prostatectomy [Article in German]. Wien Med Wochenschr. 2014;164:297–301. 24898195 10.1007/s10354-014-0264-2

[7] Lebeau T, Rouprêt M, Ferhi K, et al. Assessing the complications of laparoscopic robot-assisted surgery: the case of radical prostatectomy. Surg Endosc. 2011;25:536–542. 20614139 10.1007/s00464-010-1210-z

[8] Ghaferi AA, Birkmeyer JD, Dimick JB. Variation in hospital mortality associated with inpatient surgery. N Engl J Med. 2009;361:1368–1375. 19797283 10.1056/NEJMsa0903048

[9] Eddy DM. Clinical decision making: from theory to practice. Anatomy of a decision. JAMA. 1990;263:441–443. 2294311 10.1001/jama.263.3.441

[10] Mottet N, Bellmunt J, Bolla M, et al. EAU-ESTRO-SIOG guidelines on prostate cancer. Part 1: screening, diagnosis, and local treatment with curative intent. Eur Urol. 2017;71:618–629. 27568654 10.1016/j.eururo.2016.08.003

[11] Schroeck FR, Krupski TL, Sun L, et al. Satisfaction and regret after open retropubic or robot-assisted laparoscopic radical prostatectomy. Eur Urol. 2008;54:785–793. 18585849 10.1016/j.eururo.2008.06.063

[12] Robles L, Slogoff M, Ladwig-Scott E, et al. The addition of a nurse practitioner to an inpatient surgical team results in improved use of resources. Surgery. 2011;150:711–717. 22000183 10.1016/j.surg.2011.08.022

[13] Merhe A, Hout M, Haidar NA, et al. Is age an independent risk factor for perioperative mortality and morbidity after radical prostatectomy? Analysis of the American college of surgeons national surgical quality improvement program database. Arab J Urol. 2020. [Epub ahead of print]. DOI:10.1080/2090598X.2020.1721165. PMC7473160 33029410

[14] Sood A, Kachroo N, Abdollah F, et al. An evaluation of the timing of surgical complications following radical cystectomy: data from the American college of surgeons national surgical quality improvement program. Urology. 2017;103:91–98. 28216450 10.1016/j.urology.2017.01.036

[15] Wakeam E, Hyder JA, Tsai TC, et al. Complication timing and association with mortality in the American college of surgeons’ national surgical quality improvement program database. J Surg Res. 2015;193:77–87. 25260955 10.1016/j.jss.2014.08.025

[16] Lawson EH, Hall BL, Louie R, et al. Association between occurrence of a postoperative complication and readmission: implications for quality improvement and cost savings. Ann Surg. 2013;258:10–18. 23579579 10.1097/SLA.0b013e31828e3ac3

[17] Bilimoria KY, Cohen ME, Ingraham AM, et al. Effect of postdischarge morbidity and mortality on comparisons of hospital surgical quality. Ann Surg. 2010;252:183–190. 20531000 10.1097/SLA.0b013e3181e4846e

[18] Sood A, Abdollah F, Sammon JD, et al. An evaluation of the timing of surgical complications following nephrectomy: data from the American college of surgeons national surgical quality improvement program (ACS-NSQIP). World J Urol. 2015;33:2031–2038. 25910477 10.1007/s00345-015-1564-x

